# Proactive and Integrated Management and Empowerment in Parkinson's Disease: Designing a New Model of Care

**DOI:** 10.1155/2020/8673087

**Published:** 2020-03-30

**Authors:** Emma Tenison, Agnes Smink, Sabi Redwood, Sirwan Darweesh, Hazel Cottle, Angelika van Halteren, Pieter van den Haak, Ruth Hamlin, Jan Ypinga, Bastiaan R. Bloem, Yoav Ben-Shlomo, Marten Munneke, Emily Henderson

**Affiliations:** ^1^Department of Population Health Sciences, Bristol Medical School, University of Bristol, Bristol BS8 1NU, UK; ^2^Radboud University Medical Centre, Donders Institute for Brain, Cognition and Behaviour, Department of Neurology, Center of Expertise for Parkinson & Movement Disorders, Nijmegen, Netherlands; ^3^National Institute for Health Research Applied Research Collaboration (NIHR ARC West), 9th Floor, Whitefriars, Lewins Mead, Bristol BS1 2NT, UK; ^4^Older People's Unit, Royal United Hospitals Bath NHS Foundation Trust, Combe Park, Bath, UK

## Abstract

Parkinson's disease is the second most common neurodegenerative condition after Alzheimer's disease. The number of patients will rise dramatically due to ageing of the population and possibly also due to environmental issues. It is widely recognised that the current models of care for people with Parkinson's disease or a form of atypical parkinsonism lack continuity, are reactive to problems rather than proactive, and do not adequately support individuals to self-manage. Integrated models of care have been developed for other chronic conditions, with a range of positive effects. A multidisciplinary team of professionals in the United Kingdom and the Netherlands, all with a long history of caring for patients with movement disorders, used knowledge of deficiencies with the current model of care, an understanding of integrated care in chronic disease and the process of logic modelling, to develop a novel approach to the care of patients with Parkinson's disease. We propose a new model, termed PRIME Parkinson (Proactive and Integrated Management and Empowerment in Parkinson's Disease), which is designed to manage problems proactively, deliver integrated, multidisciplinary care, and empower patients and their carers. It has five main components: (1) personalised care management, (2) education and empowerment of patients and carers, (3) empowerment of healthcare professionals, (4) a population health approach, and (5) support of the previous four components by patient- and professional-friendly technology. Having mapped the processes required for the success of this initiative, there is now a requirement to assess its effect on health-related and quality of life outcomes as well as determining its cost-effectiveness. In the next phase of the project, we will implement PRIME Parkinson in selected areas of the United Kingdom and the Netherlands.

## 1. Introduction

Parkinson's disease (PD) is the second most common neurodegenerative disease after Alzheimer's disease. There is also a group of conditions that may initially resemble PD and that are collectively referred to as atypical parkinsonism; this includes rarer conditions such as multiple system atrophy, progressive supranuclear palsy, and Lewy body dementia. The global prevalence is estimated to have more than doubled from 2.5 million individuals in 1990 to 6.1 million individuals in 2016 [1]. Around 0.3% of the population in industrialized countries have PD, rising to 1% in those aged over 60 years [2]. Neurological diseases, including PD, are now the leading cause of disability globally [3]; PD reduces life expectancy [4], negatively impacts on health-related quality of life [5], and is associated with an increased frequency of hospital attendance at higher-than-average costs [6].

There is a growing recognition that current models of care are not optimised to care for individuals with PD or atypical parkinsonism (in the remainder of this paper, we will only refer to models for persons with PD, noting that many of the proposed solutions will likely also apply to persons with a form of atypical parkinsonism). The heterogeneity of PD necessitates a highly personalised approach whereby treatment is tailored based on patients' clinical phenotype and specific symptoms [7]. Current care models are plagued by several drawbacks: there is often a lack of continuity of care; issues are detected late and managed reactively; care is often not person-centred; and approaches may not adequately involve patients in decisions around their care [8]. Patients with PD, their relatives and healthcare professionals have previously identified several unmet needs amongst this patient group, including a need for more support to self-manage; a more collaborative approach between the multidisciplinary team and a single point of access where questions can be answered and support is given to find a way in the complex healthcare system [9].

Given the complex nature of PD, allied health involvement from appropriately skilled professionals, is paramount. Questionnaire surveys among allied health professionals, including physiotherapists, occupational therapists, and speech-language therapists who had treated patients with PD within the last year, revealed that over 75% reported a lack of PD-specific expertise [10]. In the same study, surveys of patients with PD showed that many of those experiencing problems, which would potentially benefit from therapy input, were not receiving any relevant therapy [10]. Suboptimal care appears more common for patients who are older, have worse cognition, and have mobility problems [11]. This highlights the need to improve awareness of and referral to allied health professionals and to ensure access to those with specialist expertise.

A further angle to improving care seeks to empower patients and their carers. There is an increasing awareness that patients who have the knowledge, skills, and confidence to look after their health and feel empowered to do so have better health outcomes, including being more likely to adopt healthy behaviours and attend available screening programmes, leading to improved mood and reduced rates of hospitalisation [12]. Amongst a group of patients with PD who completed the Patient Activation Measure, 42% scored as either “disengaged and overwhelmed” or “becoming aware but still struggling” [13], suggesting there is much scope to improve involvement and empowerment for people with PD.

A framework on which to base an innovative model of care was sought in order to gain an understanding of successful approaches that have been implemented and tested in other disease areas. Developed in the 1990s, the Chronic Care Model (CCM) was an approach which was designed to improve the care of people with chronic conditions and change their care from being reactive to acute events to planned and proactive [14].

Since its development, the philosophy of the CCM had positive effects on outcomes such as quality of life, functional disease status, hospitalisation rates, and adherence to guidelines, when applied to a range of different conditions, including diabetes, heart failure, chronic obstructive pulmonary disease, rheumatoid arthritis, and chronic diseases in general [15]. It can be challenging to deliver effective care to people with PD who are often older [16] and also given the complex and heterogenous nature of this neurological condition, which encompasses symptoms that span multiple medical specialisms and multiple morbidities. There is, therefore, potential to improve current care for persons with PD by incorporating the theoretical approach of the CCM which emphasises the need to support patients to self-manage; improve healthcare professionals' expertise, and utilize clinical information systems [17, 18]. A previous research project in Canada has used the “Expanded CCM,” which combines the framework of the CCM with principles of population health promotion, to develop the Chronic Care Model for Neurological Conditions (CCM-NC) [19]. However, as far as we are aware, there has only been one previous application of the CCM to the population of individuals with PD, implemented amongst US Veterans [20] and none within a European setting.

The aim of this paper is to describe the development phase of a project setting out to design a new integrated model of care, termed “PRIME-Parkinson” (Proactive and Integrated Management and Empowerment in Parkinson's Disease), for people with PD. We have chosen to also include patients with atypical parkinsonian syndromes, rather than restricting our model to patients with idiopathic PD because of the many shared healthcare needs of these patient groups, and also because we acknowledge that there is often diagnostic uncertainty, particularly in the early phases of the disease [21]. Within the context of this study, PD can, therefore, be assumed to include all forms of Parkinsonism, unless otherwise specified.

## 2. Methods

In order to develop our model, we first considered the goals of healthcare improvement, secondly the deficiencies in current PD care, and then used the process of logic modelling to design our intervention. These stages are described in further detail below.

### 2.1. Aims of Healthcare Improvement

The “Triple Aim” describes the three overarching goals which should be pursued in order to achieve improvement in healthcare systems: a desire to improve population health, improve the experience of care, whilst reducing the per capita cost of healthcare [22]. Acknowledging the risks of work-related stress and burnout, these aims were expanded by Bodenheimer and Sinsky to include the goal of improving the work-life of those who deliver care, thus creating a quadruple aim [23]. It was agreed that the new model of care should aim to reach/achieve all four aspirations described in the “quadruple aim,” acknowledging that the model would need to be at least cost neutral, even if not cost saving.

### 2.2. Identifying Issues with Current Care

The process begun with a face-to-face meeting to discuss the findings of recent qualitative work exploring the unmet needs of patients with PD [9], alongside personal experiences of delivering care to this group, in order to identify key issues with the current model of care. These were collated into a list of six main issues with current care (“the problems”) and, for each one, an accompanying statement was written to describe the collective vision for what would constitute success in this area (“the challenge”) (Table 1).

### 2.3. Rationale for Logic Model Development

We used knowledge of unmet needs of patients with PD combined with knowledge of the CCM, and its application to other chronic diseases, to design a model of care for PD. No specific author guidelines were used for the development phase. In order to graphically represent the components of the intervention, together with the theory around how each component would be expected to elicit change in outcomes, the process of logic modelling was used. Logic models enable us to consider the causal relationships between components of an intervention as well as the barriers and facilitators which may influence its success, and it enables us to build in methods of process evaluation to allow us to explain unexpected outcomes of a complex intervention [24, 25]. Logic models have been used in the development of a range of health promotion strategies including interventions to prevent childhood obesity [26], to prevent and control HIV [27], and to integrate mental health into management of chronic disease [28].

Knowledge of the CCM indicated that multiple components would need to be incorporated in order to address all the issues identified with current PD care. Having multiple components is one feature of a “complex intervention” [29], as defined by the Medical Research Council, in guidance which was updated last year [30]. It is acknowledged that evaluation of complex interventions can be challenging [31].

By clearly defining our intervention within the framework of a logic model, it is possible to build in comprehensive methods of process evaluation, alongside the evaluation of the intervention's effectiveness [24, 32]. This approach makes it possible to determine which elements of a model do or do not work, draw conclusions about how an intervention might translate to another context, and in the case of overall ineffectiveness, establish if this is due to the intervention itself or simply the way in which it was implemented [24]. We believe we have used a hybrid approach to intervention development consistent with what O'Caithan and colleagues refer to as the “theory and evidence-based” approach so that interventions are based on combining published research evidence and formal theories (e.g., organisational theories) and “target population-centred” so that interventions are based on the views and actions of the people who will use the intervention [33].

### 2.4. Approach to Logic Model Development

A group of healthcare specialists with expertise in the care of patients with movement disorders, including neurologists and geriatricians, alongside therapy, nursing colleagues, and methodologists, from both a team in the United Kingdom (UK) (Royal United Hospital, Bath) and a team in the Netherlands (Radboud University Medical Centre, Nijmegen), worked collaboratively to design a novel and integrated model of care for patients with PD, including those with PD, atypical Parkinsonian syndromes and those with cognitive impairment. The decision to include patients with all forms of Parkinsonism, with the exception of drug-induced Parkinsonism, was taken with the aim of making this care model as generalizable as possible to the day-to-day clinical practice of healthcare professionals in the field of movement disorders. Likewise, this model is intended to be applicable to patients with PD, regardless of their age or stage of disease, acknowledging that younger patients are likely to have different needs to those who are older or have additional frailty or comorbidities.

**Figure 1:**
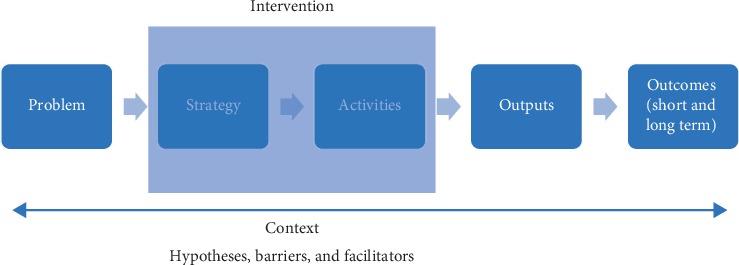
The logic model structure used to design the intervention.

**Figure 2:**
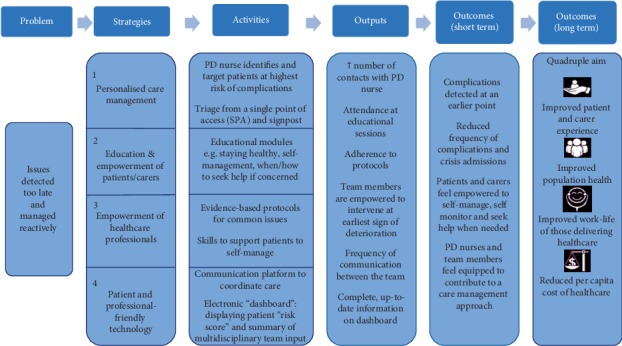
The logic model structure was applied to one of the six “problems with current care” in order to show potential strategies and activities to address the problem, the outputs/process measures, and the anticipated outcomes. This figure summarizes the content from the detailed logic model which was developed.

Having agreed upon the issues with current care, we used the structure shown in Figure 1 to define the logic model for each of the six problems shown in Table 1. This was an iterative process which entailed a subgroup of individuals, who had been involved in the project conception individually mapping out potential strategies for all six problems. These solutions were then compared and refined over a series of teleconferences, in order to achieve a unified logic model.  Figure 2 shows a simplified version of the logic model which was developed for one of the six problems: the late detection and reactive management of issues that arise in people with PD.

Having carefully mapped out the key strategies which would be expected to impact on the problems with current care, we considered the resources required to implement these changes. This led to the categorization of the strategies and activities into the five main components of our proposed model. The detailed logic model was presented back to the whole group at a face-to-face meeting four months after the process mapping had begun, and following further refinement, consensus was reached on the final logic model. We describe the main elements of our model and its overall philosophy below.

## 3. Results

We have designed a novel model of care for PD that brings together components from other chronic diseases which comprises

1. Personalised care management
2. Education and empowerment of patients and carers
3. Empowerment of healthcare professionals
4. A population health approach
5. Patient- and professional-friendly technology

The means by which these components are delivered will need to be tailored according to the country and region of care delivery. However, we propose several key elements which, together, form the basis of an integrated model of care for PD described in further detail below. [Supplementary-material supplementary-material-1] shows a summary version of the full logic model, containing these elements. The description of the components of the PRIME-Parkinson model is for illustrative purposes; we aim to publish comprehensive standard operating procedures as these are developed.

### 3.1. Personalized Care Management

A central component of the model is to arrange adequate personalized care management for every patient. Care management is the collaborative approach to organise multidisciplinary care for, and in close collaboration with, the patient and his near ones [34]. In the literature, care management and case management are used interchangeably. Case management, however, is focused mainly on supporting patients to navigate and organise their care, whereas care management refers to a broader concept [35]. It includes, alongside the direct support of patients, the integration of care among health professionals. In many cases, it is envisaged that a Parkinson's nurse will have a central role in care management.

The Parkinson's nurse facilitates collaboration between all healthcare professionals involved in a patient's care, supports patients to self-monitor and self-manage, provides information to patients and carers, and ensures that they know how to access the right help at the right time. A “single point of access” will be available for patients with PD, as well as supporting anyone else who may have concerns or questions, such as carers, the general practitioner, community teams, and secondary care; the individual who takes the call can triage the enquiry towards the appropriate multidisciplinary team member, with the assistance of the Parkinson's nurse as required. The importance of specialist nurses in caring for people with PD was acknowledged over 20 years ago in the UK, and they are recognized to fulfil several different roles, which complement those of other multidisciplinary team members [36]. These include providing practical and emotional support to patients and their carers; supporting patient education; coordination of care and acting as a link between the patient, primary, and secondary care; and lifestyle advice and goal-setting [36].

The Parkinson's nurse adopts a person-centred approach by formulating a personalized care plan; this process allows the Parkinson's nurse to establish what matters most to each patient and understand their individual needs and preferences, including for what their treatment is delivered. The Parkinson's nurse ascertains the patient's goals for treatment and assists them in setting realistic and achievable goals, together with an agreed action plan to address their goal; this information can be documented in the care plan and shared with the multidisciplinary team who can periodically enter updates and review whether the goals have been achieved and, if not, what further assistance could help with this. This collaborative sharing of information amongst the team enables the Parkinson's nurse to monitor progress on the action plan and proactively intervene if required.

Patients with PD travel along their “journey” as the disease progresses, and their needs change with increasing motor and nonmotor symptom burden. Personalised care will be ensured by accounting for PD severity and impact, especially their risk of acute deterioration and hospital admission which will allow the Parkinson's nurse to adjust their approach accordingly and proactively target those in the highest risk group. The aim is to respond to the earliest sign of deterioration in the patient's condition, ideally before they have the need to present to acute services and to instigate timely measures to stabilise the clinical and/or social situation. In situations in which acute admission is necessary, the Parkinson's nurse would be informed, enabling them to support the inpatient team to achieve continuity of care and aim to minimise the length of stay.

### 3.2. Education and Empowerment of Patients and Carers

Patients and their carers will be supported to access relevant information in a variety of formats, including written and electronic resources, group education, and peer support (both one-to-one and group support).

Given the large amount of information available on a national level, for example, as provided by Parkinson's UK [37], the goal will be to deliver personalised education by ensuring that patients are directed towards the most relevant information for them, based on their disease stage, priorities, health skills, and symptom burden and support them in both access, comprehension and relevance to their personal circumstances. The specific needs of carers will be addressed by offering opportunities for carers to share experiences, access information about financial and practical support and learn how to look after their own wellbeing.

Education will focus on common themes such as managing medication, staying healthy, self-management, and advance care planning, as well as targeting certain disease phases/stages (early diagnosis, complex disease phase). This will not just ensure understanding, in order to promote patient participation in decision-making, but also take a pragmatic approach on what actions patients might consider in being proactive rather than reactive. It is also critical that efforts to empower patients consider the changing complex nature of where patients are on their chronic disease journey. A recent review highlighted the development of five different attributes; acceptance, coping, self-management, integration and adjustment [38]. Attempts to empower patients may be problematic without understanding and accounting for these different attributes and how they are translated into ways of living.

### 3.3. Empowerment of Healthcare Professionals

We recognise a need to educate and upskill healthcare professionals, including clinicians and therapists, to ensure that they have PD-specific expertise [10] and are able to work in this new model of care by supporting them in multidisciplinary collaboration and delivery of personalised care. In addition, we will ensure that up-to-date guidelines will be made readily accessible for all healthcare professionals. Furthermore, development of evidence-based tools and protocols will standardise the approach to commonly occurring issues, focusing on the following issues in particular. These conditions have been chosen based on the large impact they have on risk of hospitalisation and mortality [39]:

- Prevention of falls and resulting fractures
- Reducing urinary tract infection
- Reducing neuropsychiatric disorders including delirium, hallucinations, and psychosis
- Reducing mood disorders and anxiety (including depression)
- Reducing pneumonia caused by swallowing issues
- Reducing social and functional decline

An online collaboration platform (see section below on technology) will allow all health professionals to see at a glance who is involved in each patient's care and what priorities have been set for each patient's care. Regular multidisciplinary team meetings will facilitate discussion of complex cases and ensure goals of treatment are aligned.

### 3.4. A Population Health Approach

A central philosophy in our model is to deliver care according to a population health approach, which can be defined as: “an approach that entails both (1) a clinical perspective, focused on delivering care to groups enrolled in a health system; and (2) a broader perspective, focused on the health of all people in a given geographic area and emphasises multi-sector approaches and incorporation of nonclinical interventions to address social determinants of health” [40]. This means that a collaborative group of healthcare professionals in a certain geographic region feel a responsibility to improve care for all patients, carers, and other people within their region. Because of this responsibility, these health-care professionals will organise and integrate care in the region and deliver joint services, e.g., information, educational courses or a helpline, to all patients with PD, carers and the wider population in the region. There is some initial evidence to suggest that this approach leads to better outcomes, while overall healthcare spending remains the same [40]. A population approach will not only consider the upstream sociocultural determinants, but will also focus on health inequalities and equitable access to health care. Whilst we may or may not be able to modify the existing sex-related differences in PD risk (men being at higher risk) [41], it is essential that all sections of society access care in relation to need and not on the basis of age, sex, socioeconomic status, ethnicity, or other factors [11]. Any new interventions should address existing inequities rather than widen them (“intervention generated inequalities”) which may occur if more educated patients are better able to make use of these interventions [42].

### 3.5. Patient and Professional Friendly Technology

We will provide a platform which can be accessed by all healthcare professionals involved in a patient's care, as well as by the patients themselves, through which to communicate securely and align and coordinate care. This platform, whilst not being a substitute for the (electronic) health record, will give an up-to-date insight into the members of the multidisciplinary team involved in a given patient's care, allocated actions, and progress on obtaining the goals set out in the personalised care plan. We envisage the development of a patient “dashboard” which enables staff to rapidly ascertain a patient's risk category, as well as showing all inputs from the multidisciplinary team. We will also support professionals with integrated decision support solutions to assist evidence-based care. We note that several efforts are currently ongoing to translate abstract plans on the role of technology in the model into concrete action points.

Some patients may benefit from monitoring their symptoms in the home environment. We will develop electronic survey-based monitoring, which was beneficial in other chronic diseases [43]. Furthermore, monitoring based on wearable devices can collect relevant information that can be communicated to a relevant healthcare professional or carer with a low burden for patients themselves. Whilst not appropriate for all, some patients may wish to access video consultation and others may benefit from being directed towards freely available, approved apps, specific to their particular needs. We plan to have smaller substudies that will look at the value of this enhanced digital technology. Emerging evidence supports this approach. A recent study demonstrated the value of home sensing devices for early detection of urinary tract infections in people with dementia [44].

### 3.6. Application of the PRIME-Parkinson Model

Table 2 shows a hypothetical case study of a patient with PD and exemplifies some of the problems which such a patient may encounter within the existing system. An alternative scenario is then suggested to demonstrate how implementation of some components of the PRIME model may have led to a better outcome.

## 4. Discussion

In the PRIME-Parkinson project, we present a new integrated model for managing PD and the various forms of atypical Parkinsonism, which incorporates five key approaches.

Whilst no evidence has yet been gathered on the utility of PRIME-Parkinson care, as a whole, it draws on previous research into specific elements of care and attempts to learn from previous positive and negative findings which relate to each of the five components of the PRIME-Parkinson model as well as responding to the challenges that both patients and professionals express around the delivery of care.

The first component of the PRIME-Parkinson model is that of personalised care management. Despite the lack of evidence to support the benefit of PD nurse specialists on clinical outcomes or quality of life, their input has a positive impact on subjective patient wellbeing, as measured with a global health question [46]. In another small study, which randomised patients to receive nurse practitioner input versus usual care, 96.2% of patients felt that provision of specialist nurses should be an important priority for the health service [47]. Indeed, guidance from the National Institute for Health and Care Excellence (NICE) states that patients need access to monitoring and a point of contact for support and information, suggesting this may be delivered by a Parkinson's nurse [48].

In the PRIME-Parkinson model, the intention is for a Parkinson's nurse to take a significant role in care management. A trial in the United States which randomised PD patients to PD nurse-led case management, including development of a personalised care plan, coordination of care, and use of communication tools, found improvement in several PD quality indicators (assessed by review of medical records and by participant survey), but concluded that work is needed to determine if this translates into improved patient-centred outcomes, such as quality of life and depression scores [49]. A randomised controlled trial in which the intervention group had access to a PD nurse specialist in the role of case manager, alongside other interventions, including regular home visits, a telephone hotline, and individual treatment plans, did find an improvement in PD-related quality of life and motor and nonmotor symptoms at 6 months [50].

The second component of PRIME-Parkinson, namely, education and empowerment of patients and carers, aims to provide patients with PD and their carers with information tailored to their needs [8]. The Patient Education Programme for Parkinson's Disease (PEPP) is an example of an intervention designed to address these needs; it consists of eight sessions delivered by trained healthcare professionals and draws on cognitive behavioural therapy techniques, covering topics such as self-monitoring, stress management, and how to be proactive in seeking out information [51]. In a randomised controlled trial, the PEPP reduced the burden of psychosocial problems for carers with a trend towards improved quality of life for patients [52]. These effects were replicated when the PEPP was evaluated in clinical practice [51]. We will face the challenges of providing different education packages for different stages of the disease as well as making these accessible for different ages, gender, and socioeconomic and ethnic groups. New technology may be helpful as animations and videos, if designed appropriately, may overcome barriers around literacy. A focus on equitable access to optimum care for all patients with PD and their family highlights the importance of a population health-based approach that goes beyond an individualistic medical model.

A core vision of our model is to deliver personalised, patient-centred care. Qualitative research, involving PD patients and their carers, has revealed a desire for these individuals to be actively involved in the decision-making process and for their values and preferences to be respected [8]. A Cochrane review of trials looking at personalised care planning interventions for a range of chronic conditions suggests a trend towards a positive effect on psychological health and ability to self-manage when a collaborative approach is used to plan care [53]. The process of a patient and clinician jointly agreeing goals, along with actions to address them, is an important aspect of developing a personalised care plan [53]. Goal-attainment scaling, a means of numerically comparing multiple, individualised outcomes which are meaningful to patients, has been used in a range of patient groups including frail, older adults [54]. A goal-oriented approach had positive effects, on both goal attainment and secondary outcomes, when applied to cognitive rehabilitation in PD dementia [55] and when incorporated into a programme of physical and occupational therapy for PD [56]. A nonrandomised trial in which the intervention group received assessment at a tertiary centre and formulation of an individual care plan, in addition to access to a network of upskilled Allied Health Professionals (ParkinsonNet), found no effect on the primary endpoint after correcting for baseline disease severity; however, any positive treatment effect is likely to have been diluted since only 73% of the recommendations made by the tertiary team were implemented in the community, highlighting the potential benefit of combining such interventions with a care manager role, to ensure the personalised care plan is followed [57].

The third component of the PRIME-Parkinson model focuses on empowerment for healthcare professionals. Given the complex range of symptoms experienced by patients with PD and the limitations of pharmacotherapy, the need to involve a range of allied health professionals in patient care is well-recognised; there has been a move away from a monodisciplinary approach, in which a clinician retains overall responsibility for patient care, with occasional referral to an allied health professional for input, towards a more integrated approach in which all professionals involved in a patient's care work collaboratively, keeping the patient at the centre of all decision-making [58]. An umbrella review of systematic reviews, looking at integrated care and its impact on hospitalisation in chronic diseases, noted that interventions which involved a multidisciplinary team component were more effective for management of specific conditions, such as heart failure, than for managing chronic disease in general, concluding that it is essential for multidisciplinary team members to have condition-specific expertise [59]. The ParkinsonNet strategy, developed in the Netherlands and first tested in 2004, sought to create a structure for the upskilling of healthcare professionals involved in caring for patients with PD, initially physiotherapists and later other allied health professionals [60, 61]. A cluster randomised controlled trial involving 16 clusters, randomised to receive ParkinsonNet physiotherapy versus usual care for 6 months, did not demonstrate a positive effect on functional status or quality of life, although did show a reduction in costs [62]. However, only one-third of patients in the intervention group received ParkinsonNet physiotherapy, therefore reducing the difference between control and active groups; additionally, training of physiotherapists occurred not long before enrolment of patients which may not have allowed sufficient time to develop specialist expertise [62]. Whilst the need for multidisciplinary team input in PD would seem clear, our model emphasizes the need for these multidisciplinary team members to receive augmented, PD-specific training and to ensure that input is coordinated and person-centred.

The PRIME-Parkinson model aims to use patient and professional-friendly technology to enhance delivery of education, promote collaboration between healthcare professionals, and facilitate coordination of care. The ParkinsonNet programme has utilised technology, in the form of online communities of healthcare professionals to facilitate continuing professional development, as well as communities of patients who share knowledge and experiences [60]. Giladi et al. describe an interdisciplinary team approach for PD in Tel Aviv, which is supported by therapists communicating and consulting electronically, as well as use of multidisciplinary team teleconferences to plan immediate interventions for patients approaching crisis [63]. A small study aiming to deliver person-centred care to underserved PD patients in Florida, by means of home visits conducted by movement disorder clinicians, used video recordings of the visit, uploaded to the patient electronic health record, to promote discussion of cases at a weekly multidisciplinary team meeting [64]. A smart phone-based Parkinson's Tracker App, developed to support self-management, has been found to improve medication adherence and patient-perceived quality of consultation when compared to usual care in a multicentre randomised controlled trial [65]. App-based technology is being used in a trial in which patients in the intervention arm will have access to a tablet-based, individualised physiotherapy training programme alongside an existing therapy programme [66]. This gives a further example of the way in which technology may support the delivery of proactive and integrated care and suggests the potential to investigate the benefit of enhanced technology in substudies embedded within PRIME-Parkinson.

Whilst some elements of the proposed model have been trialled before, with varying degrees of success, it is possible that real benefit will only be seen when the separate components are combined to form a truly integrated model of care. This hypothesis is supported by a review of systematic reviews which found that multicomponent interventions for chronic disease were more effective at reducing hospitalization and length of stay than interventions focusing on only one intervention [59]. While combining multiple components into one model of care, we propose that the exact combination and balance of each component that a patient receives should be individualised to them which reflects the essence of truly personalised care.

The PRIME-Parkinson model has a number of features of a complex intervention, as defined by the Medical Research Council [29]: multiple components, whose application will be tailored differently according to the patient profile, as well as the unpredictable way in which these elements may interact with one another when combined within the context of clinical practice. Evaluation within the framework of a logic model, as described here, reflects our commitments to robust assessment of the effectiveness of different components of the PRIME-Parkinson care model.

## 5. Conclusion

A new integrated model of care, PRIME-Parkinson, has been designed for patients with PD, using the framework of a logic model. The PRIME-Parkinson model comprises

1. Personalised care management
2. Education and empowerment of patients and carers
3. Empowerment of healthcare professionals
4. A population health approach
5. Patient- and professional-friendly technology

There is now a requirement to gather empirical data as to its effect on health-related and quality of life outcomes, as well as determining if it is cost-effective. This will be conducted in the Netherlands by means of an observational study involving four community hospitals who will receive the PRIME intervention and comparison data from other parts of the Netherlands. In the UK, there will be a pilot randomised controlled trial of PRIME-Parkinson care versus treatment as usual. Findings across the two countries will be assessed for triangulation, and if discordant, we will try to understand differences through our process measures and parallel qualitative work with patients, carers, and professionals. We envisage scope to adopt positive findings from these studies to other chronic neurological diseases.

## Figures and Tables

**Table 1:** Problems with current care model and challenges to be addressed by an integrated model of care.

**Problems with current care**	**The challenge is to:**

Lack of multidisciplinary collaboration and continuity of care Issues detected late and managed reactively Difficult to access healthcare professionals with appropriate expertise in a timely fashion Lack of empowerment and involvement for patients and carers Care not managed close to home “One size fits all” treatment and focus mostly on motor symptoms	Deliver integrated care and continuity of care Manage issues early and proactively Facilitate access to specialised healthcare professionals Educate and empower patients and carers Organise care close to home Deliver personalised care and “precision” medicine

**Table 2:** A hypothetical case study. Scenario 1 illustrates a chain of events resulting in an adverse outcome for a patient with PD. Scenario 2 offers an example of how this scenario could be managed differently with the application of the PRIME-Parkinson model. FRAX refers to the University of Sheffield fracture risk assessment tool [45].

Case study
Mrs. Ahmed is a 78-year-old lady who has had idiopathic PD for 5 years. She has recently begun to have a few “minor falls” which she has put down to “getting older.” She has noticed she sometimes feels dizzy when she stands up but doesn't like to bother her GP about it and knows it can be very hard to get an appointment. She considers mentioning it to her PD consultant when she next goes to clinic in 6 months' time but decides she won't because she believes the dizziness probably doesn't have anything to do with her Parkinson's disease.
*Scenario 1 (existing care model)*
Mrs. Ahmed begins to feel less confident going out and stops going to social activities. While taking the bins out one evening, she has a bad fall onto a concrete path, landing on her left side. Her neighbour calls an ambulance and she is taken to hospital, where she is diagnosed with a neck of femur fracture, requiring an operation. She develops a lower respiratory tract infection and postoperative delirium, which leads to a prolonged stay in the acute hospital, following which she is transferred to a community hospital for ongoing rehabilitation.
*Scenario 2 (PRIME care model)*
Remembering that light-headedness was mentioned at a PRIME-Parkinson-delivered information session she had recently attended, Mrs. Ahmed contacts the single point of access helpline to discuss her concerns about her recent dizzy spells. This information is logged in the collaboration platform and relayed to Mrs. Ahmed's Parkinson's nurse who telephones her to discuss her symptoms further and discovers that she has also begun to have a few falls as well as a number of “near misses.” The Parkinson's nurse explores the impact which these symptoms are having on her life; Mrs. Ahmed fears that she may not be able to attend her nephew's wedding next month due to her dizziness and poor balance.
Together, they agree a plan of action, with the aim of helping Mrs. Ahmed achieve her goal of attending the wedding:
(i) Blood pressure (BP) and medications are reviewed; the Parkinson's nurse suggests to the GP that he consider stopping amlodipine and Mrs. Ahmed is given advise about increasing her fluid intake, with a plan to review BP and symptoms following these changes. (ii) A referral is made to a physiotherapist, with specialist expertise in PD, who suggests a personalised exercise plan to improve her strength and balance. (iii) A referral is made to an occupational therapist (OT), with specialist expertise in PD, who visits her at home and advises some changes to reduce hazards and organises for some handrails to be installed. (iv) The Parkinson's nurse advises that Mrs. Ahmed organise an eye test at the opticians. (v) The Parkinson's nurse calculates a FRAX score, with PD included as a secondary cause of osteoporosis, and liaises with the GP regarding the result. (vi) Mrs. Ahmed is directed to Parkinson's UK patient information leaflet on “Falls and Parkinson's,” in case she wishes to read about further tips to reduce her risk of falling, and is given a leaflet on ways to improve her bone health. (vii) They agree to have a telephone appointment in 2 weeks to review how she is progressing towards her goal. (viii) The physiotherapist and OT document their input via the collaborative platform so the Parkinson's nurse is aware of the actions which have occurred ready for the telephone follow-up.

## Data Availability

No data were used to support this study.

## Supplementary Materials

Supplementary Materials Figure S1 summarizes the full logic model for the PRIME-Parkinson intervention. It displays the five strategies and a summary of the main activities for each which, together, forms the basis of the PRIME-Parkinson intervention. As described, these strategies are designed to address the six “challenges” which were identified.
