# Supplementary material for: Proactive and Integrated Management and Empowerment in Parkinson's Disease: Designing a New Model of Care
Source: Parkinsons Dis. 2020 Mar 30;2020:8673087. doi: 10.1155/2020/8673087 (PMC7149455; doi:10.1155/2020/8673087)
Supplement: Supplementary Materials — Figure S1 summarizes the full logic model for the PRIME-Parkinson intervention. It displays the five strategies and a summary of the main activities for each which, together, forms the basis of the PRIME-Parkinson intervention. As described, these strategies are designed to address the six “challenges” which were identified. [file 8673087.f1.pdf]

Figure S1: a summary of the full logic model for the PRIME-Parkinson intervention.

Challenges	Strategies	Activities
1. Deliver integrated care and continuity of care<br>2. Manage issues early and proactively<br>3. Access to specialised healthcare professionals<br>4. Educate and empower patients and carers<br>5. Organise care close to home<br>6. Deliver personalised care and "precision" medicine	**I. Personalised care management**	- Agree how members of the multidisciplinary team can contribute to a care management approach and who is leading on this for each patient.<br>- Develop and implement educational modules regarding roles and responsibilities, treatment options and tools for healthcare professionals and patients.<br>-Support professionals involved in care management to coordinate care and empower patients.<br>- Proactively target patients at highest risk of admission.<br>- Support care managers to deliver care at home or in care homes.<br>- Develop IT infrastructure to support care managers.
	**II: Education and empowerment of patients and carers**	- Detect the gaps in patients' and carers' education and empowerment.<br>- Develop and implement written / e-learning regarding the big six symptoms[1].<br>- Direct patients and carers towards the most relevant information for them, based on their disease stage, priorities, health skills and symptom burden.<br>- Facilitate peer support groups for patients and carers.<br>- Educate patients and carers regarding self-monitoring and self-management and provide tools to support this.<br>- Develop IT infrastructure to support patients.
	**III. Empowerment of healthcare professionals**	- Provide an overview of care network for each individual patient.<br>- Detect regional gaps and make consortium agreements and create networks.<br>- Develop and implement written / e-learning, regarding the big six symptoms[1].<br>- Support professionals to develop Parkinson's-specific expertise and the skills to support patients to self-manage.<br>- Facilitate multidisciplinary team meetings for discussion of complex cases.<br>- Facilitate continues learning environment and augmented training.<br>- Develop IT infrastructure to support healthcare professionals.
	**IV. Population health approach**	- Identify all stakeholders for each region.<br>- Reach consensus regarding roles and responsibilities of each of the stakeholders and the treatment options.<br>- Develop and agree to regional multidisciplinary team protocols.<br>- Sign regional agreements regarding the vision of care, the roles and responsibilities, and the treatment options with multidisciplinary team protocols.
	**V: Patient and professional friendly technology**	- Facilitate safe documentation of multidisciplinary team input and plans for follow-up.<br>- Create a platform to facilitate communication and collaboration between the multidisciplinary team.<br>- Provide infrastructure<br>* to support peer-to-peer contact or multidisciplinary team-consultation.<br>* to create a continuous learning environment for patients and healthcare professionals.<br>* for video-consultations, where relevant.<br>- Develop home-based self-monitoring IT tools.<br>- Provide helpline/single point of access.

[1] **'Big six symptoms'**: a focus on reducing/preventing the following issues:

- falls and resulting fractures
- urinary tract infection
- neuropsychiatric disorders including delirium, hallucinations, and psychosis
- mood disorders and anxiety (including depression)
- pneumonia caused by swallowing issues
- social and functional decline
